# Full-System Simulation and Analysis of a Four-Mass Vibratory MEMS Gyroscope

**DOI:** 10.3390/mi16040414

**Published:** 2025-03-30

**Authors:** Chenguang Ouyang, Wenzheng He, Lu Jia, Peng Wang, Kaichun Zhao, Fei Xing, Zheng You

**Affiliations:** 1 Department of Precision Instrument, Tsinghua University, Beijing 100084, China; ouychg.13@foxmail.com (C.O.);; 2 Engineering Research Center for Semiconductor Integrated Technology, Institute of Semiconductors, Chinese Academy of Sciences, Beijing 100084, China

**Keywords:** full-system simulation, multiphysics coupling, process-induced error compensation, MEMS gyroscope

## Abstract

This study presents a full-system simulation methodology for MEMS, addressing the critical need for reliable performance prediction in microsystem design. While existing digital tools have been widely adopted in related fields, current approaches often remain fragmented and focused on specific aspects of device behavior. In contrast, our proposed framework conducts comprehensive device physics-level analysis by integrating mechanical, thermal and electrical modeling with process simulation. The methodology features a streamlined workflow that enables direct implementation of simulation results into fabrication processes. We model a MEMS gyroscope as an example to verify our simulation approach. Multiphysics coupling is considered to capture real-world device behavior, followed by quantitative assessment of manufacturing variations through virtual prototyping and experimental validation demonstrating the method’s accuracy and practicality. The proposed approach not only improves design efficiency but also provides a robust framework for MEMS gyroscope development. With its ability to predict device performance, this methodology is expected to become an essential tool in microsensor research and development.

## 1. Introduction

The increasing demand for high-performance, cost-effective, and miniaturized devices has driven significant interest in system-level simulation techniques for MEMS devices, aiming to enhance design efficiency and device performance [1]. Over the past decades, advancements in device modeling, fabrication processes, and circuit simulation technologies have successfully addressed system-level simulation challenges for simple MEMS devices, such as MEMS pressure sensors [2]. However, complex MEMS devices, particularly gyroscopes, present unique challenges due to their inherent stiffness nonlinearity, material thermal effects, and the intricate interplay of mechanical, electrical, and thermal phenomena [3,4]. Despite progress in MEMS device development through statistical modeling, process parameter control, and experience-based predictions using operational databases [5,6], these methods remain time-consuming and costly, impeding efficient research and optimization. For MEMS gyroscopes, system-level simulations employ diverse methodologies [7], including the formulation of motion equations [8], structural optimization [9], reduced-order modeling [10], system lumped modeling [11,12], control methods simulations [13], stress analysis [14], acoustic noise evaluation [15], investigation of drive mode nonlinearity [16], orthogonal error assessment [17], and process deviations analysis [18]. While valuable, these methods are often knowledge intensive and problem specific, and require distinct simulation software. Identifying an optimal simulation framework remains a significant challenge in achieving system-level simulations of MEMS gyroscopes that align with experimental results.

MEMS technology encompasses multiple disciplines, including electronics, mechanics, materials science, manufacturing, information systems, and control theory, each supported by specialized simulation tools. For instance, the finite element simulation software like COMSOL 6.1 [19] and Ansys 20 [20] enables precise modeling of multiphysics phenomena in MEMS devices. Coventor [21] and Intellisuite [22] provide comprehensive workflows for structure-process-circuit simulations, while circuit simulators such as Cadance [23] and Simulink [24] facilitate detailed electrical analyses. Electromagnetic simulation software using CST [25] insights into signal integrity and device behavior.

Despite the availability of numerous simulation tools, selecting an appropriate framework remains non-trivial due to disparities in modeling approaches that can lead to significant variations in simulation results. Achieving comprehensive system-level validation necessitates careful selection of simulation methodologies tailored to specific physical phenomena, geometries, materials, and manufacturing processes. For MEMS devices, no universal solution exists that can accommodate all design requirements.

To address these challenges, we present a case study on the system-level modeling and analysis of a MEMS four-mass gyroscope, integrating electromagnetic performance simulations, fabrication process modeling, parameter analysis, and error characterization. Our approach achieved successful system-level validations with experimental results closely matching simulation predictions. This research advances MEMS modeling by providing a validated framework that bridges theoretical simulations with practical manufacturing considerations, offering valuable insights for device optimization and microsystem technology development.

## 2. Method

**Figure 1:**
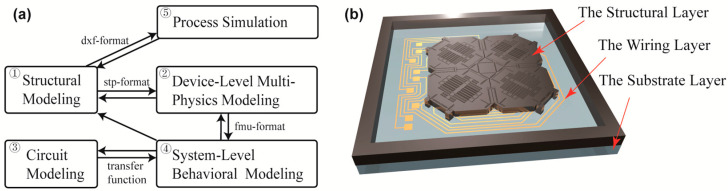
(**a**) Procedure of the system-level simulation; (**b**) model of the gyroscope structure.

The full-system simulation framework, as shown in Figure 1a, comprises the following steps. (1) 3D device structural modeling: Parametric modeling using SolidWorks 2022 to generate STEP-format files. Establishment of constraint relationships between parametric dimensions (e.g., parallelism, symmetry) to create configurable models. (2) Multiphysics modeling: Import 3D models into COMSOL for comprehensive dynamic, electrical, thermodynamic, and coupled mechanical–electrical analyses to assess device performance under various operating conditions. Iterative structural optimization of key parameters (e.g., beam width impact on resonant frequency) to achieve target performance. (3) Develop a circuit model, derive its transfer function, and integrate it into Simulink for co-simulation. (4) Export reduced-order model (FMU) to Simulink for co-simulation with circuit models. Simulate sensor electrical response under physical stimuli (angular velocity → capacitance variation → voltage output). Design optimization prioritizes MEMS structural adjustment (e.g., comb finger design) over ASIC modifications due to cost/time constraints. (5) Process Simulation: Generate 2D process layouts from 3D structures for manufacturing simulation. Perform fundamental design rule checks: layer alignment, photoresist (positive/negative) selection, process compatibility, line width/spacing verification. This systematic approach enables a holistic understanding of the MEMS device’s behavior under realistic operating conditions, ensuring accurate predictions for performance optimization.

In the structural modeling step, the 3D model of the mechanical structure is constructed. The gyroscope system consists of two main elements: the MEMS sensing structure and associated circuit. As illustrated in Figure 1b, the MEMS sensing structure comprises three layers, including the structural layer, the wiring layer, and the substrate layer. The structural layer generates variations in capacitance in response to external angular velocities, while the wiring layer is responsible for conveying electrical signals. Moreover, the substrate provides a robust foundational platform that supports the entire system architecture.

Table 1 provides a list of variables utilized in the system modeling, accompanied by their respective explanations. For the sake of brevity and clarity, these variables will be directly referenced in the subsequent formulas without additional description. The working principle of a resonant gyroscope can be described as follows: under the influence of the drive force, the sensitive mass block oscillates along the drive direction. When angular velocity is applied along the sensitive axis, the Coriolis effect induces a motion of the sensitive mass in the sensing direction. The displacement caused by this motion is detected via changes in comb capacitance, enabling the calculation of the angular velocity. The gyroscope operates as a typical second-order damped system, and its motion equation can be simplified as

(1) $$\left[\begin{array}{c} \ddot{x}\\ \ddot{y} \end{array}\right]+\left[\begin{array}{cc} \omega_{x}/Q_{x} & 0\\ 0 & \omega_{y}/Q_{y} \end{array}\right]\left[\begin{array}{c} \dot{x}\\ \dot{y} \end{array}\right]+\left[\begin{array}{cc} \omega_{x}^{2} & 0\\ 0 & \omega_{y}^{2} \end{array}\right]\left[\begin{array}{c} x\\ y \end{array}\right]=\left[\begin{array}{c} f_{d}\sin\left(\omega_{d}t\right)/m\\ 2\Omega_{z}\dot{x} \end{array}\right]$$

By setting $\omega_{d}=\omega_{x}$, the solution of above equation can be expressed as:

(2) $$x\left(t\right)=\frac{Q_{x}f_{d}}{m\omega_{x}^{2}}sin\left(\omega_{x}t+\varphi_{x}\right)=A_{x}sin\left(\omega_{x}t+\varphi_{x}\right)\;y\left(t\right)=\frac{-2\Omega_{z}f_{d}Q_{x}}{m\omega_{x}}\frac{1}{\sqrt{{\left(\omega_{y}^{2}-\omega_{x}^{2}\right)}^{2}+\omega_{y}^{2}\omega_{x}^{2}/Q_{y}^{2}}}sin\left(\omega_{x}t+\varphi_{y}\right)$$

The mechanical sensitivity is given by:

(3) $$S_{y}=|\frac{y\left(t\right)}{\Omega_{z}}|=|\frac{2f_{d}Q_{x}}{m\omega_{x}}\frac{1}{\sqrt{{\left(\omega_{y}^{2}-\omega_{x}^{2}\right)}^{2}+\omega_{y}^{2}\omega_{x}^{2}/Q_{y}^{2}}}|$$

At the condition where $\omega_{x}=\omega_{y}$ the sensitivity reaches its maximum value:

(4) $$S_{ymax}=\frac{2f_{d}Q_{x}Q_{y}}{m\omega_{x}^{3}}$$

The drive and sensing modes play a critical role in determining the overall performance of the sensor. A higher mechanical sensitivity directly translates to an improved SNR for the system, while simultaneously reducing the required driving force.

A decoupled symmetric four-mass structure is proposed for this MEMS gyroscope. The inherent symmetry of the design enables robust angular rate measurement that is minimally affected by extraneous variables such as ambient temperature fluctuations and common-mode noise interference, thereby significantly enhancing the device’s reliability and anti-interference capabilities. This makes it particularly suitable for applications requiring precise and high SNR operation in demanding environments [26]. The four-mass structure operates based on a configuration where four lumped masses are distributed symmetrically around a circumference. These sensing masses oscillate in an anti-phase motion pattern, enabling effective differential signal extraction. The structure’s distinct four-fold symmetrical axes endow it with an operating principle akin to that of Hemispherical Resonant Gyroscopes (HRGs), demonstrating its potential for achieving navigation-grade performance with excellent precision and stability [27]. The decoupling mechanism between the drive and sensing modes effectively mitigates coupling error impacts caused by manufacturing tolerances [28,29].

### 2.1. Device-Level Multiphysics Modeling

In the model analysis step, we considered the modal order, nonlinear capacitance, tuning stiffness, drive mode nonlinearity, quality factor, mechanical thermal noise and wiring and signal flow simulation.

(1) Modal order

**Figure 2:**
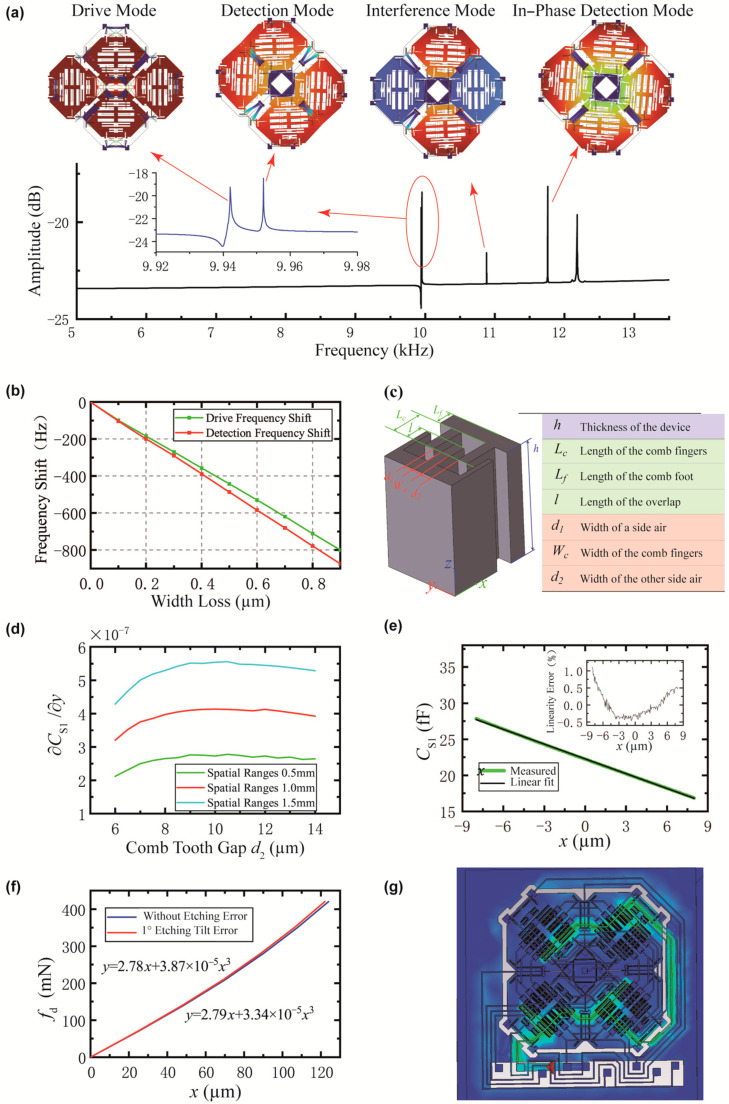
(**a**) Amplitude–frequency response showing the first five modal peaks; (**b**) relationship between linewidth loss and modal resonance frequency for drive (green) and sensing (red) modes; (**c**) model of a parametric comb structure with movable and fixed teeth; (**d**) relationship between comb finger gap and capacitance sensitivity across different spatial ranges; (**e**) simulation of driving capacitance vs. displacement with linear fitting and linearity error; (**f**) driving force vs. displacement in driving direction with and without 1° etching tilt error; (**g**) complete signal analysis with routing and structure, visualizing driving voltage flow.

In actual operation, the frequencies of the drive mode and sensing mode are carefully aligned to achieve maximum operational sensitivity. However, due to manufacturing tolerances, the design intentionally sets the sensing mode frequency slightly higher than that of the drive mode. Electrostatic tuning techniques are then employed to reduce the sensing mode frequency until it matches the drive mode frequency [30]. As shown in Figure 2a, the drive mode is configured as the first-order mode, while the sensing mode is set as the second-order mode, with the sensing mode frequency being 10 Hz higher than that of the drive mode.

The drive and sensing modes are designed as low-frequency anti-phase resonant modes, with the relative sensing mass moving in opposite directions with equal amplitudes. This configuration effectively suppresses common-mode vibrations, thereby enhancing the sensor’s sensitivity to motion in a specific direction. In contrast, the in-phase mode is prone to external accelerations, which may degrade performance. However, in conventional designs, the in-phase mode frequency is lower than that of the anti-phase mode, making it more susceptible to interference from environmental noise. Due to manufacturing tolerances, in-phase motion can couple into the anti-phase motion, as described by the following equation:

(5) $$\begin{array}{l} x_{an}+\omega_{an}^{2}x_{an}=\frac{\Delta K}{2m}x_{in}+\frac{F}{2m}\\ x_{in}+\omega_{in}^{2}x_{in}=\frac{\Delta K}{2m}x_{an} \end{array}$$

where $x_{an}$, $x_{in}$, $\omega_{an}$, $\omega_{in}$ and m are shown in Table 1, F represents the actuating force, and ΔK refers to the stiffness error induced by manufacturing tolerances.

The in-phase sensing mode is tuned to a frequency approximately 2 kHz higher than the anti-phase sensing mode as shown in Figure 2a. The third-order mode is spaced more than 800 Hz apart from both the drive and sensing modes, while the in-phase drive mode is tuned to a frequency more than 5 kHz higher than the anti-phase drive mode.

Linewidth loss refers to systematic deviations between fabricated MEMS feature dimensions (e.g., beams, trenches) and their design specifications, primarily caused by non-ideal photolithography and etching processes. This manufacturing effect directly impacts performance metrics such as resonant frequency. This effect was identified through numerical simulations and is demonstrated in Figure 2b. A clear relationship exists between line width loss and frequency offsets for both drive and sensing modes. Specifically, the drive mode exhibits a frequency offset of approximately 971 Hz/μm, while the sensing mode shows an offset of approximately 889 Hz/μm. These losses cause an additional increase of 82 Hz/μm in the frequency difference between the drive and sensing modes. Therefore, process compensation must be carefully integrated into design-stage optimization to account for specific fabrication conditions. In this study, process-induced errors were not quantified during the initial design phase, resulting in the lack of explicit process compensation strategies.

(2) Capacitance sensitivity and nonlinearity

Comb capacitance can be categorized into two configurations: variable spacing and variable area types. As illustrated in Figure 2c, the behavior of moving comb structures depends on their direction of motion. Specifically, combs that move along the x-direction correspond to the variable area type, while those moving along the y-direction represent the variable spacing type. The capacitance of a comb structure is given by $C=\varepsilon hl(\frac{1}{d_{1}}+\frac{1}{d_{2}})$. According to the principle of virtual work $F=\frac{1}{2}\frac{𝜕C}{𝜕x}U^{2}$, the static forces along the x/y direction of movement are,

(6) $$F_{x}=\frac{1}{2}\varepsilon h\left(\frac{U_{1}^{2}}{d_{1}}-\frac{U_{2}^{2}}{d_{2}}\right)\;F_{y}=\frac{1}{2}\varepsilon hl\left(\frac{1}{(d_{1}-d_{y}{)}^{2}}-\frac{1}{(d_{2}+d_{y}{)}^{2}}\right)\left(U_{1}^{2}-U_{2}^{2}\right)$$

where $U_{1}$ and $U_{2}$ are the applied voltages, ε is the dielectric constant, h, l, $d_{1}$ and $d_{2}$ represent the height, overlapping length, and the spacing between the two sides of the comb, respectively, and $d_{y}$ is the displacement of the moving comb teeth in the y-direction.

To enhance capacitance sensitivity, the design employs a variable spacing comb structure with biased combs ($d_{1}\neq d_{2}$). Considering the actual fabrication constraints, we set $d_{1}=4\;µm$ ($d_{1}<d_{2}$), while maintaining fixed values for the comb width and overlap length of 7 µm and 60 µm, respectively. The capacitance sensitivity can be optimized by adjusting the comb gap $d_{2}$ in different spaces, as demonstrated in Figure 2d. Specifically, setting $d_{2}=10\;µm$ significantly improves the comb sensitivity.

In the drive direction, high linearity must be achieved to simplify control systems. Therefore, variable-area combs are employed. As demonstrated in Figure 2e, simulations show that the nonlinearity remains below 1% at a drive displacement of 8 µm.

(3) Drive mode nonlinearity

Increasing the drive force ($f_{d}$) is capable of improving the mechanical sensitivity. However, the force-displacement nonlinearity causes frequency-amplitude hysteresis, thereby degrading the overall gyroscope performance [16,31]. Conversely, optimizing the trade-off between sensitivity and nonlinear effects can enhance device performance. The force-displacement nonlinearity of the gyroscope is characterized by a cubic term, $F(\begin{array}{c} x) \end{array}=k_{1}x+k_{3}x^{3}$, where the gyroscope drive frequency is [32],

(7) $$\omega =\omega_{x}+\frac{3k_{3}}{8k_{1}\omega_{x}}A_{x}^{2}$$

where $A_{x}$ represents the amplitude of the drive displacement, and $\omega_{x}$ is the frequency of the drive mode.

The structural nonlinear simulation is shown in Figure 2e, and the cubic term coefficient of the nonlinearity is $0.0334\;\mu N/{\mu m}^{3}$. Non-vertical etching can transform a rectangular beam into a trapezoidal beam, leading to an increase in nonlinearity. Based on the prior manufacturing results, the etching verticality error does not exceed 1°, the coefficient of the cubic term increases to $0.0387\;\mu N/{\mu m}^{3}$, which is an increase in approximately 15%.

(4) Electrostatic tuning

The tuning comb-electrodes are designed as biased combs of the variable area type ($d_{1}\neq d_{2}$). The electrostatic force stiffness and frequency shift are given by,

(8) $$K_{s}=\frac{𝜕F}{𝜕y}=\varepsilon hl\cdot {∆U}^{2}\left[\frac{1}{{\left(d_{1}+d_{y}\right)}^{3}}+\frac{1}{{\left(d_{2}-d_{y}\right)}^{3}}\right]\approx \varepsilon hl\cdot {∆U}^{2}\left(\frac{1}{d_{1}^{3}}+\frac{1}{d_{2}^{3}}\right)\;\omega_{y}=\sqrt{\frac{K-K_{s}}{m}}$$

where ε is the dielectric constant, $∆U=U_{1}-U_{2}$ is the voltage difference across the comb, h, l, $d_{1}$ and $d_{2}$ represent the height, overlapping length, and the spacing between the two sides of the comb, respectively, as shown in Figure 2b, $d_{y}$ is the displacement of the moving comb in the y-direction, $\omega_{y}$ is the sensing mode frequency, K is the sensing mode stiffness, and $K_{s}$ is the tuning stiffness.

(5) Quality factor and mechanical thermal noise

The quality factor is inversely proportional to damping, which primarily includes air damping, anchor point damping, and thermoelastic damping. Under vacuum conditions, thermoelastic damping is the primary damping source.

(9) $$\frac{1}{Q}=\frac{1}{Q_{AIR}}+\frac{1}{Q_{ANCHOR}}+\frac{1}{Q_{TED}}$$

Derived by integrating the effects of mechanical displacement and thermal expansion under specific boundary conditions, considering factors such as beam dimensions and material properties. The analytical solution for the thermoelastic quality factor of a rectangular beam is [33],

(10) $$Q_{TED}={\left(\frac{c_{p}\rho }{E\alpha^{2}T}\right)}^{2}\left(\omega \tau +\frac{1}{\omega \tau }\right)\;\tau ={\left(\frac{b}{\pi }\right)}^{2}\frac{c_{p}\rho }{\kappa }$$

where b is the beam width, $c_{p}$ is the constant-pressure specific heat capacity, ρ is the material density, κ is the thermal conductivity, ω is the resonant frequency, E is the modulus of elasticity, α is the coefficient of thermal expansion, and T is the absolute temperature.

The complex beam structure of the gyroscope makes it challenging to establish an analytical model for thermoelastic damping. However, within the design range, narrower beam widths lead to higher quality factors. The simulated thermoelastic quality factor is $1.2\times 10^{5}$ for the drive mode and $160\times 10^{5}$ for the sensing mode.

Mechanical thermal noise, caused by molecular thermal motion in the sensing direction, is defined as,

(11) $$\Omega_{th}=\frac{abs\left(y_{noise}\right)}{abs\left(y\left(\Omega =1{}^{\circ}/s\right)\right)}=\frac{90\omega_{x}}{\pi Q_{x}f_{d}}\sqrt{\frac{4mk_{B}T\omega_{y}}{Q_{y}}}$$

where $k_{B}\;$ is the Boltzmann constant and T is the absolute temperature. When the amplitude of the drive displacement is 2 μm and the quality factor is 5000, the mechanical thermal noise of this structure at room temperature is less than 0.1°/h.

(6) Wiring and signal flow simulation

The comb finger signals are connected to metal pads via metal lines, which are subsequently wire-bonded to the ASIC. Both the ASIC and the device employ single-sided routing in their design, ensuring that corresponding interfaces are directly connected. This configuration minimizes signal transmission noise and parasitic capacitance but enhancing wiring density. The four mass blocks feature evenly distributed comb finger interfaces, though improper wiring arrangements may lead to signal crosstalk, potentially degrading the measurement accuracy of comb finger capacitance.

To validate the wiring’s feasibility and assess transmission quality, electromagnetic signal simulations were conducted in CST Studio Suite. As illustrated in Figure 2g, signal injection occurred at the drive and carrier electrodes’ interfaces (green indicates signal flow). The signals propagated sequentially via metal lines to the drive comb fingers of each sensing mass block. Minimal crosstalk was observed between metal lines, confirming an efficient wiring configuration. However, electromagnetic coupling occurred when signals passed below the comb fingers. To mitigate this effect, increasing the anchor height and expanding the gap between traces and comb finger structures were implemented. Based on prior experience, an anchor height of 20 µm was selected to optimize performance.

### 2.2. System-Level Behavioral Modeling

**Figure 3:**
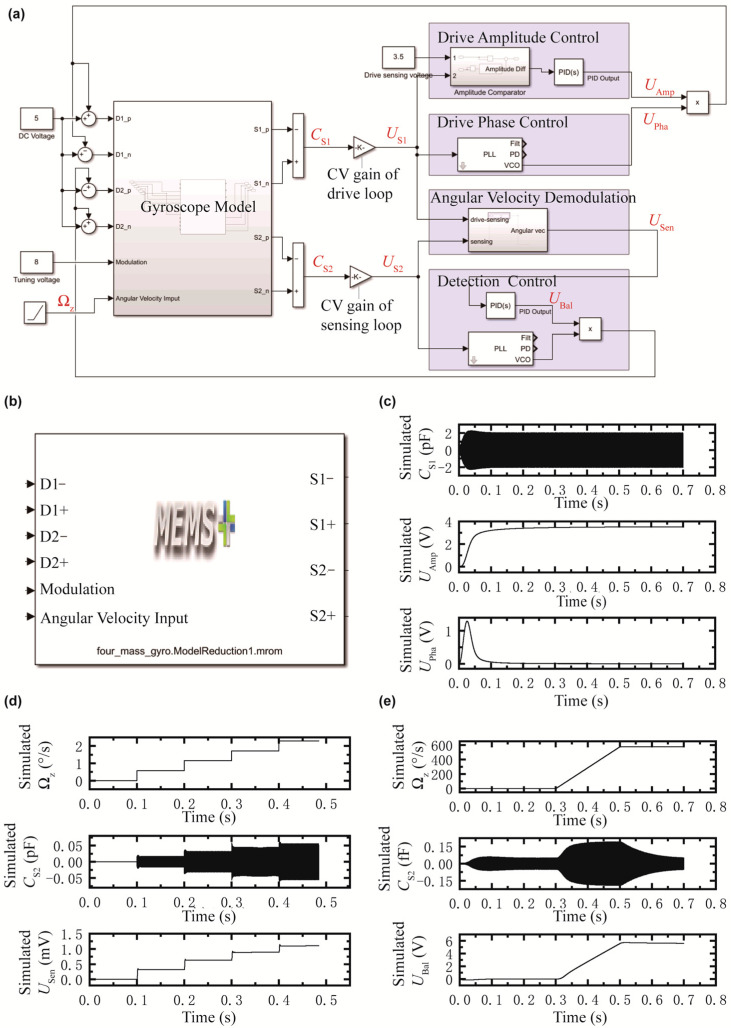
(**a**) System-level Simulink model comprises circuit model and reduced-order gyroscope sensing structure model; (**b**) reduced-order model of gyroscope sensing structure with input (left side) and output (right side) signal interfaces; (**c**) closed-loop drive simulation results showing the time variation in drive-sensing capacitance, amplitude control voltage, and phase control voltage (top to bottom); (**d**) open-loop sensing simulation results showing the time variation in angular velocity input, sensing capacitance change, and demodulated angular velocity signals; (**e**) closed-loop sensing simulation results showing the time variation in angular velocity input, sensing capacitance change, and force-balance voltage.

In this study, physical-level circuit models were constructed using component libraries from TINA, followed by transfer function derivation. These functions were then imported into Simulink to establish system-level behavioral models. The system-level simulation results are shown in Figure 3 (see Table 1 for a complete list of symbols), incorporating gyroscope sensing structure model and circuits model for comprehensive system analysis, as illustrated in Figure 3a. The circuit architecture consists of two distinct feedback loops: the drive closed loop, responsible for phase-locked and amplitude control of the drive signal; and the sensing closed loop, which performs similar operations on the sensing signal.

The reduced-order model of the gyroscopic sensitive structure is presented in Figure 3b, with input signals including positive/negative drive electrodes, positive/negative force-balance electrodes, carrier interfaces, and angular velocity inputs. The output consists of positive/negative drive-sensing electrodes and positive/negative sensing electrodes. As shown, sinusoidal drive signals of identical frequency but opposite phase are applied to the positive and negative drive electrodes, generating a driving force in the designated direction. The differential signal between the drive-sensing electrodes exhibits a sinusoidal waveform at the same frequency as the drive signal. When the drive voltage frequency matches the drive mode frequency, the system enters resonance, resulting in a 90-degree phase shift between the drive and drive-sensing signals.

The transient response of the drive closed loop is demonstrated in Figure 3c, where both amplitude and phase stabilization occur within 0.2 s. The drive-sensing signal stabilizes at 2.0 pF, and the amplitude control voltages is 3.5 V with a capacitance-to-voltage (CV) gain of 1.75 V/pF. These results confirm effective operation of the drive closed loop.

For angular velocity sensing, Figure 3d illustrates the demodulation process when an angular velocity input is applied. The differential signal between the sensing electrodes produces a sinusoidal waveform at the same frequency as the drive signal, with an amplitude proportional to the input angular velocity. A step input of angular velocity generates a corresponding step response that trends consistently with the input. At low angular velocity inputs, the system exhibits approximately linear behavior with a sensitivity of approximately 0.50 mV/(°/s). The demodulation results, obtained with a CV gain of 20 V/pF, confirm accurate angular velocity measurement within this configuration.

The force-balance is implemented by applying sinusoidal signals of the same frequency but opposite and in-phase to the positive and negative force-balance electrodes. This generates a balancing force that counteracts the Coriolis force, thereby suppressing motion along the sensing axis and reducing the amplitude of the sensing signal. As shown in Figure 3e, a ramp input of angular velocity is used for the force-balance closed-loop simulation. The results demonstrate effective suppression of the sensing signal amplitude while maintaining proportionality between the force-balance signal and the angular velocity input. This confirms that the force-balance system accurately reflects angular velocity changes and maintains equilibrium with the Coriolis force, achieving a sensitivity of approximately 9.8 mV/(°/s).

### 2.3. MEMS Process Simulation

**Figure 4:**
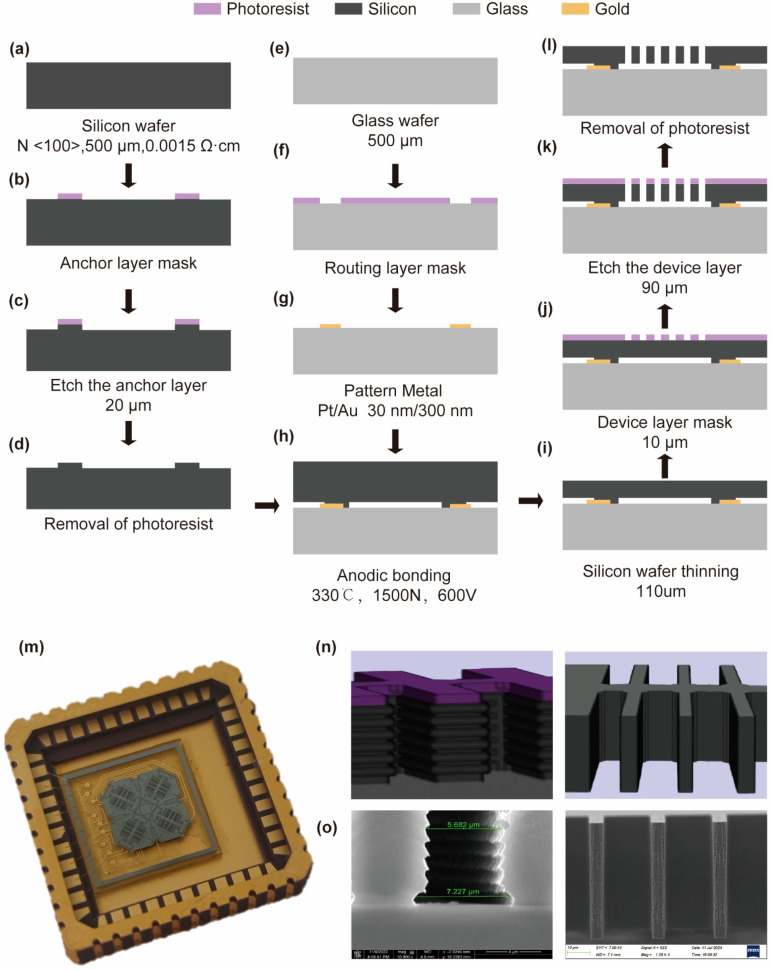
(**a**–**l**) Detailed process of SOG fabrication, including deposition and etching parameters; (**m**) completed MEMS device with wire bonding to ceramic package; (**n**) simulation showing side wall scalloping, footing, and ideal comb finger profiles; (**o**) SEM images comparing simulated and actual etching results.

The wafer-level silicon-on-glass (SOG) technology used in the fabrication of the gyroscope sensitive structure is illustrated in Figure 4. A 4-inch N-type 500 µm-thick silicon wafer with an orientation of <100> and a resistivity of 0.0015 Ω·cm, is utilized (Figure 4a). The anchor pattern is transferred onto the wafer through a photolithography process involving spin-coating with approximately 4 μm of photoresist (Figure 4b). Subsequently, deep reactive ion etching (DRIE) is applied to etch the patterned wafer and reduce its thickness by 20 μm (Figure 4c), followed by removal of the photoresist mask (Figure 4d).

A 4-inch glass wafer with a thickness of 500 μm is used (Figure 4e). The wiring pattern is transferred onto the glass through a photolithography process (Figure 4f). Subsequently, sputtering of 300 Å of platinum and 3000 Å of gold is performed. The metal wiring is finally formed by a lift-off process (Figure 4g).

The alignment and bonding of the prepared silicon wafer and glass wafer are carried out using the anodic bonding method, at a temperature of 330 °C, a pressure of 1500 N, and a voltage of 600 V (Figure 4h). The bonded wafer is then thinned by immersion in potassium hydroxide solution until the thickness of silicon layer reaches 110 μm (Figure 4i). The device patterns are then transferred onto the silicon wafer through a photolithography process, which involves spin-coating approximately 10 μm of photoresist onto the device layer (Figure 4j). To release the moveable structures, DRIE is employed to etch the bonded wafer until the silicon layer is completely etched through (Figure 4k). Finally, a plasma asher is used to remove the photoresist from the silicon layer (Figure 4l).

The processing results are shown in Figure 4m. Process simulation can check the compatibility of process steps and simulate the alignment of overlay marks. Through simulation, a qualitative understanding of process parameters and process errors can be obtained, which helps in the actual processing and debugging. Figure 4n presents the simulation of sidewall etching error and undercut, as well as the processing results of the comb fingers. Figure 4o shows the corresponding processing results, which are consistent with the simulation.

## 3. Results

**Figure 5:**
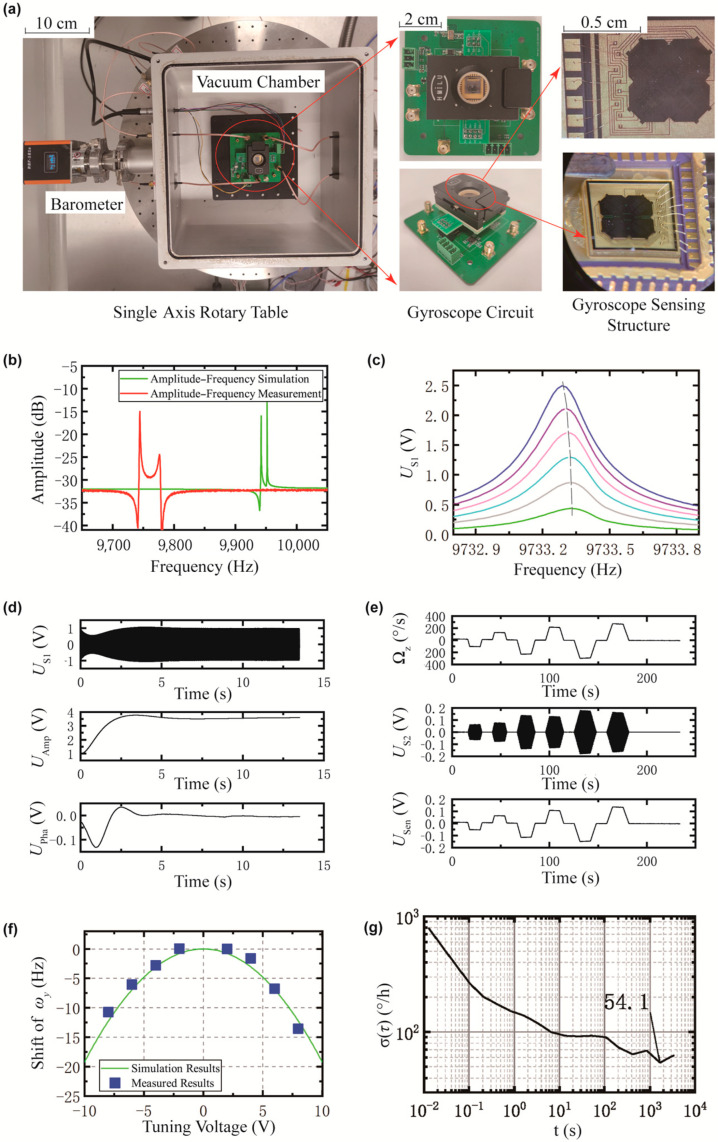
(**a**) Gyroscope testing environment including vacuum chamber and test circuit; (**b**) gyroscope amplitude–frequency response comparison, measurement (red) vs. simulation (green); (**c**) gyroscope drive mode amplitude–frequency responses under different drive force, showing drive mode frequency and drive displacement relationship. The capacitance/displacement of drive mode is 2.0 pF/μm and the drive-loop CV gain is 1.75 V/pF; (**d**) closed-loop drive test results showing the time variation in displacement sensing voltage, amplitude control voltage, and phase control signals (top to bottom); (**e**) open-loop sensing test results showing the time variation in angular velocity input, sensing voltage change, and demodulated angular velocity signals; (**f**) relationship between tuning voltage and sensing mode frequency; (**g**) Allan variance of the gyroscope.

Experimental results are shown in Figure 5 (see Table 1 and Figure 3a for a complete list of symbols). As illustrated in Figure 5a, the circuit was placed inside a vacuum chamber fixed on a single-axis turntable. Pressure within the vacuum chamber was monitored using a pressure gauge. The test circuit was connected externally via coaxial cables, with the gyroscope sensitive structure inserted into the chip socket.

A frequency-sweep signal was then applied to the drive comb fingers. Sensing signals were received from both the drive-sensing and sensing electrodes. Displacements in the drive and sensing directions were calculated to obtain the amplitude–frequency response, as shown in Figure 5b. Comparison with the simulation results from Figure 2a, the drive mode frequency was found to be 198 Hz lower than the simulated value, while the sensing mode frequency was 176 Hz lower. Furthermore, the frequency difference increased from 10 Hz to 32 Hz.

The width of the 108 beams in the device under test was characterized via SEM measurements, with three points sampled per beam. The average line width loss was determined to be approximately 0.2 ± 0.1 µm. Based on the established relationship from simulations between line width loss and frequency shift, the drive frequency is expected to experience a reduction of approximately 194 Hz, while the sensing frequency would decrease by approximately 178 Hz. Consequently, the frequency difference is projected to increase by 26 Hz, a result that aligns well with experimental measurements.

The relationship between the drive displacement and drive frequency is illustrated in Figure 5c. At a drive displacement of 0.7 μm, the drive frequency exhibits a variation of approximately 0.04 Hz, indicating a weak dependence of the displacement on frequency. This observation indicates minimal nonlinear effects within the drive system. The device exhibited stable performance across the operational pressure range (3–10 Pa), maintaining a quality factor ($3.6\times 10^{4}$) that remained relatively insensitive to pressure variations, thereby enhancing control accuracy. It is worth noting that the measured quality factor was significantly lower than the simulated value due to limitations in vacuum pump performance, which precluded achieving lower pressures.

The drive closed-loop control results, as shown in Figure 5d, confirm stable system behavior under normal operating conditions. The gyroscope oscillates with the drive-sensing signal amplitude maintained at 1 V. Both amplitude and phase controls achieve stable states within approximately 8 s. The drive amplitude control achieves a steady-state value of 3.5 V, providing sufficient stroke margin to prevent potential mechanical collisions. Meanwhile, the drive phase control attains a steady-temperature value of 0 V, ensuring that the phase difference between the drive and drive-sensing signals remains at 90°.

During open-loop sensing, the gyroscope’s response to turntable rotation was recorded, as shown in Figure 5e. The measured sensitivity (0.55 mV/°/s) closely matches the simulated value (0.50 mV/°/s), demonstrating strong agreement between theoretical predictions and experimental results. As shown in Figure 5f, tuning voltage-induced frequency shifts were recorded across different operating conditions, with a tuning voltage of 8 V producing a frequency shift capability of 12 Hz. However, this value is insufficient to meet the required tuning range of 32 Hz.

Static data collected over 90 min yielded an Allan variance analysis, as shown in Figure 5g, revealing a bias instability of 54°/h. In the experiment, the process compensation was not implemented, resulting in frequency deviations exceeding the tunable range, reduced mechanical sensitivity, and relatively high noise levels.

## 4. Discussion

Model accuracy is a significant challenge for designers. The coupling of design, fabrication, and circuit issues ultimately affects the final signal output. In our system model, we can separately calibrate MEMS structures (e.g., MEMS processing line width loss) and circuit models (e.g., gain factor) based on actual measurement results. This decouples design issues and system errors, allowing designers to focus on MEMS design. Establishing a cross-domain parameter feedback closed-loop simulation system is the core of our research.

To address the nonlinear coupling of structural, circuit, and process parameters in MEMS design, our simulation framework supports bidirectional parameter transfer. Building on the forward flow from the multiphysics-system behavioral process, the system also supports reverse triggering of structural parameter optimization (e.g., comb finger adjustment) based on circuit behavioral simulation results (e.g., sensitivity), forming a dynamic closed loop.

Through the decoupling and calibration of MEMS and circuit issues, along with parameterized iteration, we reduce the number of iterations. Calibration driven by actual measurement data enables the decoupling and identification of MEMS process errors (e.g., resonant frequency shift) and circuit parameter deviations (e.g., amplifier gain error). Based on Solidworks constraints, when designers adjust comb finger parameters (e.g., overlap length), the COMSOL model automatically updates, followed by interface circuit optimization in Simulink, achieving cross-domain parameter co-evolution.

After one fabrication cycle, we verified the device model, and corrected the circuit model and process errors. The simulation and experimental results are compared in Table 2. The simulated modal frequencies demonstrate strong agreement with experimental measurements, achieving estimation errors below 2%. Notably, the implementation of process compensation further reduces frequency deviations to <10 Hz, enabling precise frequency tuning. While reduced-order model of gyroscope sensing structure and simplification of circuit model significantly enhance simulation efficiency, they may also introduce distortions of system representations The sensitivity and tuning frequency estimations exhibit deviations of approximately 10%, these values remain within an acceptable range for circuit design purposes. The larger discrepancies observed in drive nonlinearity estimation still provide preliminary reference value. These deviations may originate from multiple sources beyond sidewall non-verticality errors, including line-edge roughness and structural defects introduced during DRIE.

## 5. Conclusions

In this study, we present a systematic framework for analyzing a four-mass gyroscope device through hierarchical multi-level simulations. This approach includes three hierarchical levels of analysis: device-level performance characterization, process-level layout validation, and system-level functional characterization. At the device-level, our simulations yielded performance metrics such as resonant frequencies, quality factors, and sensitivities. The process-level analyses validated the device layouts and fabrication processes, ensuring compatibility with the intended design specifications. Finally, the system-level analysis characterized operational parameters, including driving voltage and sensitivities.

The experimental validation of our simulation methodology has demonstrated both its accuracy and reliability. This approach provides a powerful tool for analyzing a wide range of MEMS devices, enabling effective design optimization and test circuit development while significantly reducing reliance on time-intensive laboratory experiments and associated fabrication costs.

Future research will focus on advancing the integration of topology optimization techniques with simulation-based computational modeling to further enhance device performance. Our upcoming work aims to transition from manual adjustments toward an automated optimization framework. Computational models will be used to quantify key performance metrics such as modal distributions, quality factors, and gyroscopic sensitivities across critical geometric parameters like beam widths and comb finger spacings. This systematic approach will pave the way for more efficient design iterations and improved device performance in MEMS-based gyroscope applications.

## Figures and Tables

**Table 1:** List of symbols.

Symbol	Description	Unit
x, y	The displacements along the drive and sensing motion direction.	m
$\omega_{x}$, $\omega_{y}$	The resonant frequencies of the drive and sensing modes.	rad
$x_{an}$, $x_{in}$	The displacements of the anti-phase and in-phase motion.	m
$\omega_{an}$, $\omega_{in}$	The frequencies of the anti-phase and in-phase modes.	rad
$Q_{x}$, $Q_{y}$	The quality factors of the drive and sensing modes.	/
$\Omega_{z},a$	The external angular velocity and acceleration.	rad/s, m/s^2^
$f_{d}$, $\omega_{d}$	The amplitude and frequency of the drive force applied.	N, rad
m	The resonant mass.	kg
*K*	The stiffness of resonant mode.	N/m
$C_{S1}$, $C_{S2}$	The change values of the drive-sensing and sensing capacitor.	F
$U_{S1}$, $U_{S2}$	The voltages of $C_{S1}$ and $C_{S2}$ after capacitance-to-voltage conversion.	V
$U_{Amp}$, $U_{Pha}$	The amplitude control and phase-locked control voltages in closed-loop drive.	V
$U_{Sen}$, $U_{Bal}$	The angular velocity demodulated signal voltage and force-balance voltage.	V

**Table 2:** Comparison of simulation and experimental results.

Parameter Name	Simulation Results	Experimental Results	Deviation
Drive mode frequency	9931 Hz (9737 Hz after compensated)	9733 Hz	−2.0% (−0.04%)
Sensing mode frequency	9941 Hz (9763 Hz after compensated)	9765 Hz	−1.8% (0.02%)
Thermoelastic quality factor of drive mode	$1.2\times 10^{5}$	$>3.6\times 10^{4}$	/
Sensitivity (open-loop)	0.50 mV/°/s	0.55 mV/°/s	10%
Tuning frequency	12.7 Hz (8 V)	11.8 Hz (8 V)	−7.1%
Drive frequency drift	0.022 Hz (0.7 μm) 0.025 Hz for 1° etching verticality error	0.040 Hz (0.7 μm)	60%

## Data Availability

No new data were created.
